# Elective Umbilical Hernia Repair in Adults in the 21st Century: Challenging the Status Quo

**DOI:** 10.3390/jcm14176324

**Published:** 2025-09-07

**Authors:** Sergio Huerta, Jared McAllister, Crystal Phung, Angela A. Guzzetta

**Affiliations:** VA North Texas Health Care System, Dallas, TX 75216, USA; jared.mcallister@va.gov (J.M.);

**Keywords:** ventral hernia, epigastric hernia, incisional hernia, Richter’s hernia

## Abstract

On the spectrum of complexity for general surgery operations, umbilical hernia repair (UHR) is on the light side. After inguinal hernias, they are the most commonly repaired hernias and, as such, umbilical hernias are an important component of a general surgery practice. Since the time at which WJ Mayo published his seminal technique on the repair of umbilical hernias, multiple strategies for the management of umbilical hernias have emerged ranging from watchful waiting to open repair, as well as minimally invasive approaches. The present perspective maintains that each approach has its merits depending on the patient, surgeon, and institution. However, randomized controlled trials and clinical practice guidelines have favored some approaches over others. Similarly, recommendations have been developed regarding body mass index classification as well as hernia size for mesh placement. Other factors important to UHR are the choice of anesthesia and smoking cessation for elective repair. Though we do not contest well-designed randomized controlled trials (RTCs), or clinical guidelines, we offer our perspective on the care of these common hernias.

## 1. Introduction

**Figure 1:**
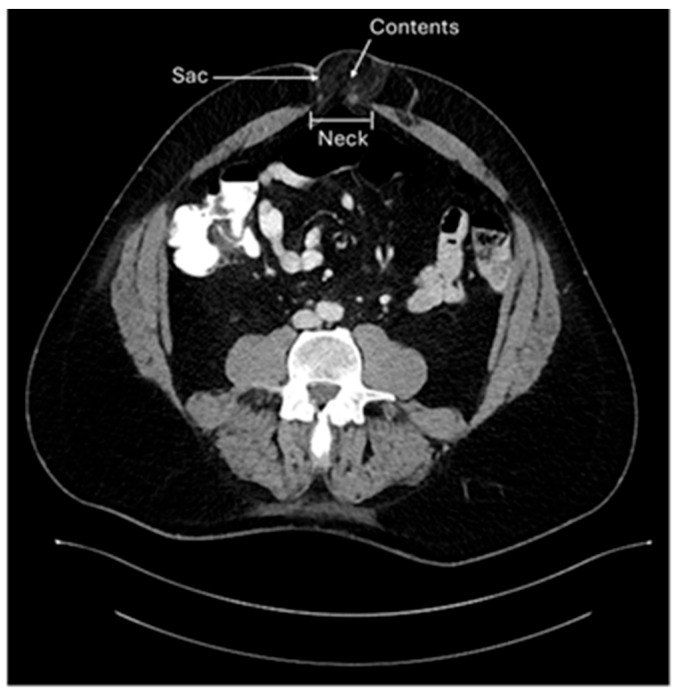
Umbilical hernia with three components: neck, sac, and contents.

A hernia occurs in an area of the body that is devoid of striated muscle. Structures normally contained within a normal compartment protrude through a “rupture” (Latin for hernia) into a non-anatomic cavity. Three components are characteristic of hernias: the neck is the rupture of the fascia that allows the protrusion of hernia contents; the hernia sac, a lining of peritoneum through the defect; and the hernia contents, which are any structures contained within the hernia sac (Figure 1) [1].

### Umbilical Hernia vs. Primary Epigastric Hernia vs. Incisional Umbilical Hernia

**Figure 2:**
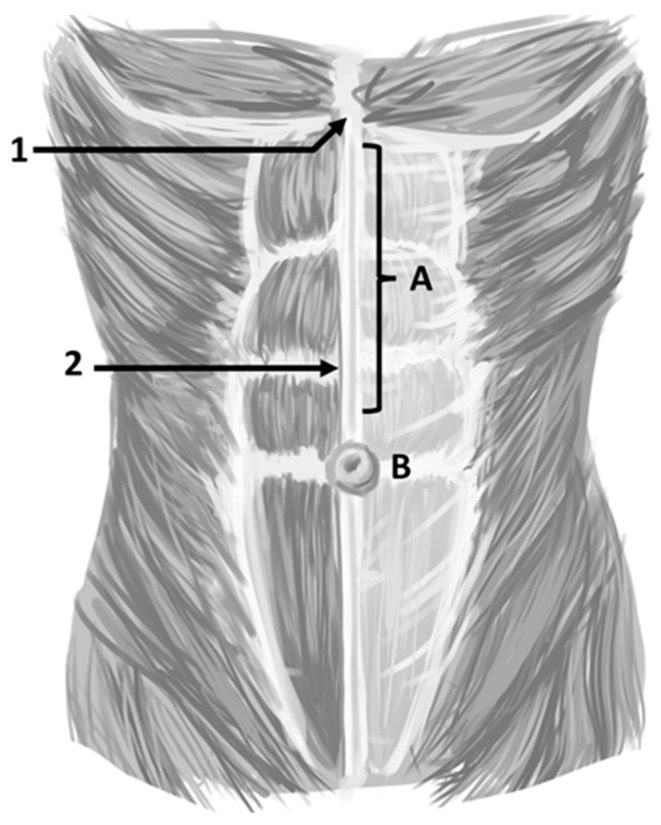
Location of umbilical hernias and epigastric hernias. A = epigastric hernia, B = Umbilical hernia. An incisional hernia can occur anywhere after an operation. 1 = Xyphoid process, 2 = linea alba. (Figure created by C. Phung).

While there is a wide range of ways to define an umbilical hernia (UH), the most logical version is the one presented by the guidelines for the treatment of umbilical and epigastric hernias from the European and Americas Hernia Societies (EHS/AHS). The EHS/AHS define an UH as a primary ventral hernia with the center of the defect located in the midline in the center of the umbilical ring. This is to be contrasted with a primary epigastric hernia, defined as a ventral hernia in the midline above the umbilicus and below the xyphoid process (Figure 2) [2]. This definition should be universally adopted for consistency and management of patients with an umbilical hernia compared to epigastric hernias. An incisional hernia might be located anywhere including the midline and the umbilical ring, but this occurs after operative interventions (e.g., placement of a trocar during a laparoscopic cholecystectomy). These distinctions are important as the authors maintain that the management and outcomes of primary epigastric and incisional hernias are different from UH. This manuscript addresses the management of UH exclusively.

**Figure 3:**
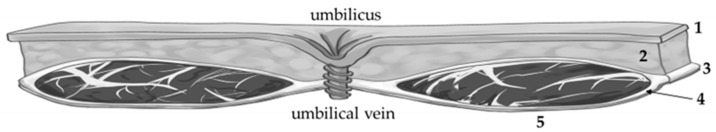
Horizontal section through the umbilicus. 1 = skin, 2 = fat, 3 = rectus sheath, 4 = transversalis fascia, 5 = peritoneum. (Figure created by C. Phung).

The linea alba is a fibro-aponeurotic structure in the middle of the abdomen formed from the coalescence and interlacing of the sheaths of the abdominus recti bilaterally. The linea alba adjacent to the umbilicus is inherently weak as this is an area that penetrates the abdominal wall for passage of the umbilical vessels as well as the urachus to the placenta during fetal development. Neither the superficial fascia, nor the transversalis fascia, nor the peritoneum enter in the formation of the linea alba (Figure 3). Around the umbilical ring the surrounding fascia is particularly strong [3].

Umbilical hernias in adults have their origin in early infancy [3]. In umbilical hernias, the transversalis fascia is pierced by the blood vessels that pass through the umbilicus. Theoretically, it is possible to have an UH through an opening of the right umbilical artery; a hernia through the opening of the left umbilical artery; a hernia through the opening of the allantois (urachus); or an UH through the opening of the umbilical vein. Practically, the most common type of UH is the one that is through an opening of the umbilical vein (Figure 3).

The pathophysiology of primary epigastric hernias is different in that the linea alba outside of the umbilical ring is inherently weak. The anterior abdominal wall forms in the first 60 days of gestation. The muscles and aponeuroses grow from the dorsal surface starting at 33–37 days gestation. By 57 days gestation, the aponeurosis meets at the midline ventral surface [4]. Closer examination of the midline demonstrates irregular crossings of fibers at the midline. The linea alba has a wide variety of thicknesses but thickness directly correlates with burst strength [5].

The weakness of the linea alba is further compromised by the reduced amount type I collagen and relatively increased amount of elastin [6,7]. One theory for the development of primary epigastric hernias proposes that weakness at the linea alba between the umbilicus and the xiphoid process was created by vascular lacunae formed when small blood vessels penetrated it, which left a small space where preperitoneal fat from the falciform ligament herniates and leads to enlargement [8]. Another theory maintains that epigastric hernias occur as a result of the lack of triple lines of decussation [9,10].

Regardless of the pathophysiology of an umbilical, epigastric, or incisional hernia, the following factors have been recognized to increase the likelihood of developing an acquired hernia: Intra-abdominal pressure augmentation results in constant dilation of the borders around the umbilical ring [11]. Risk factors for the development of an UH include pregnancy [11], obesity [12], ascites [13], and malignancy [14].

Author’s Practice: It is important to clearly define UHs as purely UHs versus primary epigastric hernias versus incisional hernias even if they occur anatomically in near proximity to the umbilicus. The type of repair and outcomes are dependent on the type of hernia. Purely umbilical hernias are likely to have better outcomes compared to the other types. Further, because hernia size does not affect our management, we do not typically require size classification. However, the classification of hernias as proposed by the European Hernia Society (EHS) should be undertaken to standardize surgical practice in the management of UH [2,15].

## 2. Epidemiology

Umbilical hernias in children occur in 15–23% of newborns [16]; these are the only hernias that most commonly do not require surgical intervention as they spontaneously close. Thus, watchful waiting is recommended until age 4–5, independent of the size of the hernia [17].

Umbilical hernias occur more frequently in women than men. Because of an increase in intrabdominal pressure, pregnancy might demonstrate more clearly a pre-existing hernia at the umbilical ring or lead to one de novo. Among pregnant women UHs occur frequently [18].

In obese patients, laparoscopic opening pressure increases by 0.07 mm Hg, directly proportional to a BMI increase of 1.0 kg/mm^2^ [12]. Higher BMI leads to a higher strain on the umbilical ring resulting in a higher likelihood of UH development. For instance, for a patient with a BMI of 30–39 kg/m^2^, the odds ratio of developing an UH is 2.6 compared to patients with a normal BMI and as high as 5.2 if the BMI exceeds 60 kg/m^2^. Additionally, obese patients with an existing UH are more likely to present with acute or chronic incarceration [19].

Similarly, up to 20% of patients with liver cirrhosis develop UHs. In these patients, if a liver transplant is not expected to occur within three months, elective UHR is recommended if there is acceptable functional liver reserve [20].

An incisional UH is an iatrogenic hernia. Because of the astronomic increase of laparoscopic and robotic cases in general surgery, more incisional umbilical hernias are likely to be seen in the future. The umbilicus is a common site for placement of laparoscopic and robotic trocars with 10 mm trocars or larger being the most likely culprits of incisional hernia development [21]. This is complicated by the fact that an UH might be present and unidentified at the time of the placement and removal of a laparoscopic trocar at or around the umbilicus [22]. Scar tissue has inherently lower tensile strength providing a risk factor for herniation [23].

Author’s Practice: It is our practice to routinely avoid placing any ports in the midline. Instead, we make small adjustments to place ports where abdominal musculature will help obscure any defects in the fascia to prevent herniation.

**Figure 4:**
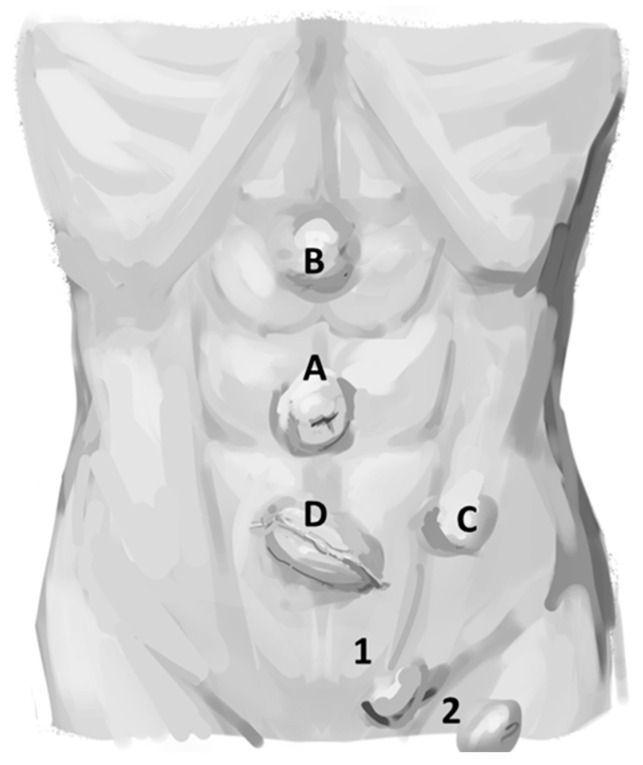
Abdominal wall hernias. A = umbilical hernia, B = epigastric hernia, C = Spigelian hernia, D = incisional hernia. A, B, C, and D might be all grouped at umbilical hernia. 1 = inguinal hernia, 2 = femora hernia. (Figure created by C. Phung).

The incidence of purely acquired UH in adults is unclear because umbilical hernias, epigastric hernias, incisional hernias, and others (e.g., Spigelian hernias) are commonly grouped together (Figure 4). Small UH are unlikely to cause symptoms, and these go underreported in the literature. The coding for the procedure is variable and data is difficult to collect retrospectively from outpatient as well as inpatient settings [24]. Similarly, the number of UHR in the United States is uncertain but UHR is regularly cited as the second most common type of abdominal wall hernia repair (after inguinal hernia repair) at 255,000 in 2003. But the number of UHR has fallen recently and is now estimated to be approximately ~1,897,290 [22]. However, another small study has demonstrated an incidence of UH in up to 23–50% of patients during physical examination aided by sonography in patients who did not come with complaints of an umbilical hernia [24]. These findings underscore the difficulties in collecting precise information regarding the true incidence of umbilical hernias.

Author’s Practice: Independent of true incidence, UHs are the second most common abdominal wall hernias and are inescapable in general surgery practice. There are many special cases of UH that have a wide variability of outcomes. For instance, the outcomes of postpartum UHR on young women are different than UHR outcomes in patients with cirrhosis, even though these operations are coded similarly. Pivotal to the academic analysis of outcomes of UH is the need to distinguish a purely UH from other special cases. This manuscript focuses entirely on the repair of purely UH, simply referred to as UH going forward.

## 3. History and Physical Examination

The history and physical examination of UH is relatively uncomplicated. Because this is less likely to lead to controversial issues, it will not be presented in this perspective manuscript, but the reader can find these items in other publications [2,22,25,26]. Umbilical hernias are asymptomatic in the absence of incarceration or strangulation. Acute incarceration with strangulation should be treated as an urgent or emergent case depending on the circumstances [2,22,25,26]. Whether in the acute setting or in the presence of chronicity, UH containing bowel must be addressed more cautiously compared to those incarcerated with fat.

Similarly, diagnostic imaging is less prone to controversy in UH. However, sonography and computed tomography (CT) are excellent studies depending on the resources of the hospital. More information regarding indications and the need for diagnostic imaging can be found elsewhere [2,22,25,26]. However, diagnostic imaging is more useful and indicated in the acute setting compared to UH presenting in outpatient clinical practice.

Author’s Practice: We typically do not require any form of diagnostic of study to offer repair of an UH. More recently, many patients have been referred to clinics with an UH and imaging obtained by primary care physicians. Some patients might present with a chronically incarcerated hernia. We treat these patients similarly to those without incarceration if there is no history of pain or obstruction, or tenderness on physical exam. Patients with morbid obesity and a thick abdominal wall may benefit from preoperative imaging because their exams can be misleading.

## 4. Management

**Figure 5:**
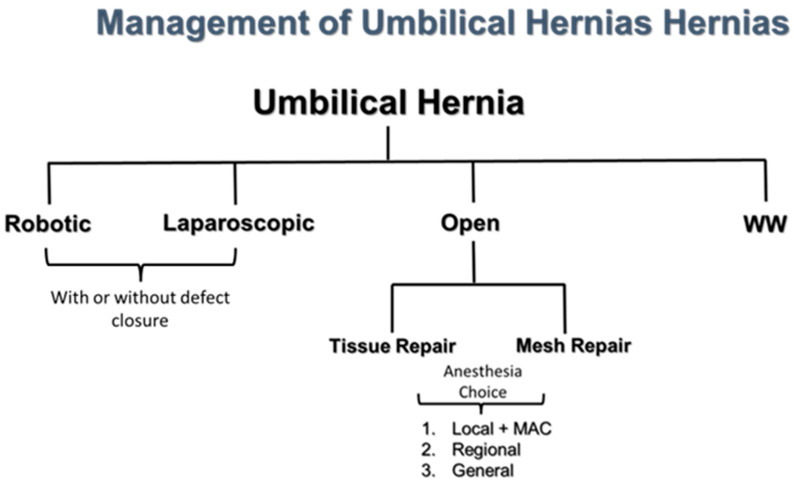
Management of Umbilical Hernias in the 21st Century. WW = watchful waiting. MAC = monitored anesthesia care.

The management of UH has a wide spectrum with watchful waiting (WW) on one side and robotic repair on the other (Figure 5). While we recommend WW in patients with asymptomatic UH and a high risk for an operation, it should be noted that unlike asymptomatic inguinal hernias, there are no trials to suggest that WW of UH is safe. In primary ventral hernias, defects that were 3–4 cm were significantly associated with bowel incarceration [27]. Because primary epigastric hernias and incisional hernias are more likely to increase in size over time or become symptomatic, these are less likely to be offered WW. The management of choice is dependent on the patient (degree of comorbidities), the experience of the surgeon (open vs. laparoscopic; mesh vs. primary repair), the institution (no laparoscopic or robotic platforms available), and the choice of the patient [requesting local and monitored anesthesia care (MAC)]. In our institution, we have a team that can offer each one of these modalities of repair and anesthesia choices.

Author’s Practice: This type of management is not intended to suggest that one technique is superior to another. In fact, we suggest that we should not view a variety of strategies as competing, but complementary. Further, system-based practices must be considered.

## 5. Local Anesthesia for Umbilical Hernia Repair

A major advantage of the open technique for UHR is the ability to perform it under local anesthesia (LA) and monitored anesthesia care (MAC) [LA + MAC]. This is particularly advantageous for older patients with a burden of comorbid conditions; the only option for UHR may be utilizing LA + MAC [28].

For inguinal hernias, there is substantial evidence to demonstrate the LA + MAC is superior to other forms of anesthesia in terms of outcomes and cost [29]. For UHR, the evidence is less clear as there have not been any RCTs comparing LA + MAC vs. general anesthesia or LA + MAC vs. regional anesthesia [30]. The European Hernia Society and the American Hernia Society indicate that there is currently no evidence to support the superiority of LA over GA for UH [2]. Two systematic reviews for UH under LA have shown this to be a feasible approach, especially in frail patients [28,30,31]. A feasibility study at our institution (*n* = 53) found that veteran patients submitting to open UHR under LA + MAC had a higher American Society of Anesthesia (ASA) class and an increased rate of cardiovascular disease. Despite this, 30-day complication was reduced in the LA + MAC cohort by univariable analysis. After correcting other variables including hernia size, the complication and recurrence rates were similar by multivariable analysis. Operative room time was significantly reduced by univariable analysis in the LA + MAC cohort. LA + MAC patients did not require post-anesthesia care unit (PACU) recovery time, which led to a savings of over 50 min compared to the general anesthesia cohort [32].

Despite strong evidence that LA + MAC is superior to other forms of anesthesia for inguinal hernias, it is not widely practiced in the United States. This is primarily guided by surgeon’s preference for general anesthesia [33]. In low- and middle-income countries (LMICs), regional anesthesia is preferred because of cost and convenience. These are the same factors that dominate the use of general anesthesia over LA + MAC for UHR in the United Sates and for regional anesthesia in LMICs.

Author’s Practice: In our practice of older veteran patients with a substantial burden of comorbid conditions, we prefer to use LA + MAC for most open UHR. The only contra-indication for this practice is patient’s preference.

## 6. Indications for Elective Repair and Surgery

This section addresses a few controversial issues in offering repair of UH in the elective setting: BMI and a history of smoking as well as primary repair instead of the commonly viewed approach to utilizing mesh for most patients. This section is not intended to dispute the evidence of RTCs, society guidelines, or even well-designed retrospective reviews. We offer this perspective based on the repair of over 600 umbilical hernias at the VA North Texas Health Care System with over 20 years of experience by the primary author of this manuscript (SH).

### 6.1. Mesh Versus Primary Repair

There is overwhelming evidence in favor of mesh over tissue repair for the management of UH to reduce recurrence [2,34,35,36,37]. Mesh placement for UHR has been documented in systematic reviews, RCTs, and multiple observational studies. A systematic review of mesh vs. tissue repair indicated that using mesh resulted in a 10-fold decrease in recurrence without affecting the complication rate, which included surgical site infections (SSI) [38]. Historical recurrence without mesh has been up to 54% [39,40,41]. More recent studies demonstrate recurrence with mesh is as low as 0% to 3% [40,42,43,44] and 14% for suture repair [45,46]. Thus, RCTs, systematic reviews, and clinical guidelines [2,25] almost universally advocate for mesh placement for almost all UH to prevent recurrence.

The opinions presented in this perspective do not dispute this evidence. However, we believe that tissue repair does not violate standards of care [47,48,49]. This is predicated by the fact that multiple centers (including ours) still perform high numbers of tissue repair for primary UH [46,50,51,52,53,54,55,56]. Multiple retrospective studies have indicated equivalent recurrence in mesh repair compared to tissue repair of UH [57]. Our own studies have shown no difference in the rate of recurrence with mesh (3.0%) compared to tissue repair (7.7%, *p* = 0.14) [52]. Additionally, other authors have revealed no difference in recurrence in UH that were smaller than 3 cm if mesh was used compared to no mesh [54]. Another observational analysis of 392 patients with UH demonstrated no difference in recurrence in tissue vs. mesh repair [58]. The authors in these analyses have argued against mesh because of an increase rate of potential complications including an increase in SSI (19.0% to 19.8 with mesh vs. 7.9% to with tissue repair) [38,43,58]. Additionally, while umbilical mesh repair is common in North America, reports from Sweden and Denmark demonstrate primary UHR in 70% to 77% of their cases [54,59].

Arguments against mesh implantation emanate from potential complications ranging from 6.0% to 42.0% [46,60]. Proponents of tissue repair suggest a balance in the rate of recurrence with the complication burden and that the best method to accomplish this is not well established. In our practice, tissue repair for UH has many advantages: (1) decreased risk of SSI [61,62,63], (2) decreased risk of mesh migration [64], (3) reduced operative times [28], (4) performance more readily under LA + MAC, [30] and (5) an ability to proceed posteriorly if there is a recurrence with an anterior approach.

The advocacy of mesh placement over tissue repair is much stronger in obese patients and in umbilical hernias with large neck size.

#### 6.1.1. Mesh Versus Primary Repair Based on BMI

Hernia size and BMI have been suggested to determine when mesh is indicated to prevent recurrence of UHR. At this juncture, there have been no RCTs to address specific BMI and hernia size cutoffs for mesh placement. Many studies have suggested the use of mesh for patients with a BMI > 35 kg/m^2^ [22,35,65]. Because obesity might predispose a patient to develop a SSI, the EHS/AHS guidelines recommend weight loss in obese patients prior to elective repair [2].

**Figure 6:**
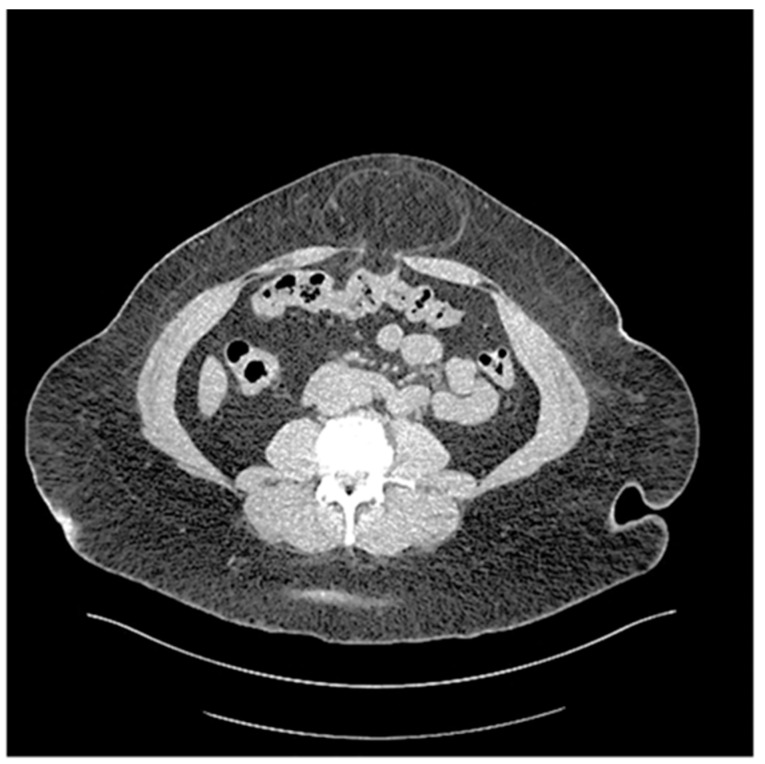
Chronically incarcerated Umbilical hernia in a patient with a BMI 39.3 kg/m^2^.

The rationale for placing mesh in obese patients (BMI ≥ 30 kg/m^2^) with UH is that these patients have relatively higher intraabdominal pressure compared to non-obese patients, thus increasing recurrence risk as well as incarceration (Figure 6).

However, we maintain that primary UH repairs can be performed in obese patients. In two separate studies at our institution, we found no correlation between BMI and recurrence [52,53]. One previous study in our veteran patient population found two recurrences in 47 patients with a BMI of 35–39.9 kg/m^2^. The same study identified only one recurrence in 14 patients with a BMI > 40.0 kg/m^2^ who underwent elective UHR [53]. This indicated that primary tissue repair of UH is feasible even among patients with high BMIs.

Our analysis demonstrated a low rate of recurrence with this approach and no difference in rates of recurrence between obese and non-obese patients. This is in agreement with other studies that have investigated factors that lead to an UH recurrence and have not found obesity to be linked with recurrence [43,46,52,53,66]. In our investigation, we had 199 obese patients who underwent UHR under primary tissue repair by a standardized technique by the same surgeon (SH). All patients were from the same institution and had an average follow up of 3.8 ± 0.15 years. In this observational study, the recurrence was 4.0%. Postoperative complications occurred in 9.0% of patients. We found no difference in the rate of recurrence in obese vs. non-obese patients. Further, univariable analysis did not find obesity to be associated with recurrence. Finally, multivariable analysis did not show obesity as an independent risk factor for recurrence [66].

Further studies have found that obese patients who are asked to lose weight prior to repair of an UH are unable to do so and may present in the acute setting with an incarceration [67]. Complications are always higher in the urgent/emergent setting compared to elective repair [68]. Our experience is similar to other reports, and we support elective primary umbilical hernia repair in obese individuals who are unable to lose weight or refuse bariatric surgery prior to repair [47,48,49,52,53,69]. However, it cannot be ignored that evidence contrary to these findings also exists in the literature [51,70].

#### 6.1.2. Mesh Versus Primary Repair Based on Hernia Neck Size

A neck size of 2.0 cm was codified by many textbooks as a cut-off number to justify mesh placement [46,55,56,71,72]. A study by Schumacher et al. [55] systematically analyzed UH neck size and recurrence to recommend mesh placement. This small study (*n* = 108) showed a recurrence rate of 6.3%, 4.1%, 14.3%, 25.0%, and 54.5% for UH size of <1.0 cm, 1.0 cm to 2.0 cm, 2.0 cm–3.0 cm, 3.0 cm to 4.0 cm, and >4.0 cm, respectively. Based on these findings mesh placement was recommended for UH defects > 1.5 cm [55]. In a separate study, hernias were separated according to the following sizes: 1.0 cm to 1.5 cm, 1.5 cm to 3.0 cm, 3.0 cm to 4.0 cm, and >4.0 cm. This study included mesh and tissue repair. No difference was found in the recurrence rate independent of size between tissue and mesh repair. Regardless, the authors recommended mesh repair for hernia size > 2.0 cm [54]. In a larger study (*n* = 949) Donovan et al. compared mesh (50.9%) to primary repair (49.1%) and found that hernias >1.5 cm repaired primarily was an independent predictor of recurrence [51]. There was no difference in recurrence based on the use of mesh versus primary repair [51]. Other studies suggested a 1.0 cm to 1.5 cm size defect cutoff for mesh placement [35,36,50,51,55,73,74,75]. Today, RCTs and guidelines advocate for mesh placement to reduce recurrence for almost all hernia sizes [76].

In a study by our group, 359 veterans underwent UHR by a standardized open tissue repair technique by the same surgeon (SH) [66]. The recurrence rate was 4.7%, and multivariable analysis did not demonstrate an association between hernia size and recurrence. A similar analysis was undertaken by Kang et al. that also controlled for surgeon in 231 primary UHRs. The authors found a significant difference in recurrence rates based on size (2.0 cm vs. 1.4 cm; *p* < 0.05). Primary epigastric hernias and incisional hernias may benefit from mesh placement or laparoscopy [77]. This indicates that hernia size influences but does not prohibit acceptable recurrence rates for tissue repair with a standardized technique.

Author’s Practice: In our practice a primary repair might be undertaken independent of BMI or hernia neck size provided that the tissue comes together without tension. The decision of mesh placement is made in the operating room if there are other hernias or the tissue has undue tension. However, the literature firmly indicates that mesh repair remains the preferred approach, recommending tissue repair for small defects, patients with a high infection risk, and patient’s preference refusing the use of mesh.

#### 6.1.3. Transverse Versus Vertical Primary Repair of Umbilical Hernias

The original umbilical hernia repair by Mayo in 1901 described a transverse closure to minimize tension resulting from Valsalva as the oblique muscles contracted [78]. He also argued that this closure would allow proper approximation of larger defects. The transverse approach since this publication has been codified in textbooks as the “gold-standard”.

Author’s Practice: In my (SH) 20-year practice of repairing over 600 umbilical hernias in veteran patients, my approach has been to close these hernias vertically. I use 1-0 PDS in an interrupted fashion, after I have cleared the edges of the fascia well and entirely reduced the hernia sac.

#### 6.1.4. Elective Umbilical Hernia Repair and Current Smoking History

Multiple studies have cited current smoking as a prohibitive factor to proceed with elective repair of UH [50,79,80,81,82,83]. Patients who smoke might have a three-fold increase in recurrence compared to non-smokers [83]. Another study found by propensity score matching analyses current smokers have an increased likelihood of 30-day mortality (OR 1.42), overall morbidity (OR 1.39), wound (OR 1.40), respiratory (OR 1.14), or cardiac morbidity (OR 1.88) compared to non/ex-smokers (*p* < 0.05 for all) [84]. A meta-analysis showed that smokers had significantly higher rates of recurrence (10.4% vs. 9.1%; RR 1.48; 95% CI [1.15; 1.90]; *p* <  0.01), surgical site occurrences [13.6% vs. 12.7%; RR 1.44; 95% CI [1.12; 1.86]; *p* <  0.01), and SSI (6.6% vs. 4.2%; RR 1.64; 95% CI [1.38; 1.94]; *p*  <  0.01) following VHR [85].

Theoretically, because smoking is a modifiable risk factor, patients who smoke should be asked to cease this practice prior to proceeding with elective repair. The EHS/AHS guidelines recommend a 4-week period of smoking cessation prior to elective repair [2]. However, a study in Demark found that only one fifth of patients presenting for elective repair of hernias stopped smoking prior to surgery [86]. A large percentage of our veterans have been smoking for several decades, and most are unable to stop smoking prior to elective hernia repair [87]. Furthermore, studies show a higher rate of recurrence or complications with smokers vs. non-smokers, but this difference, while statistically significant, is neither astounding nor prohibitive. Further, in the 21st century, in high-income countries, respect for a patient’s autonomy has significantly shaped the way healthcare interventions are discussed and delivered. This phenomenon has influenced medical decision-making and has led to the development of many healthcare policies [88].

Author’s practice: Our practice is to counsel patients regarding the higher risk of recurrence and smoking. We provide all the tools to help them cease smoking prior to elective repair. We give them a period of time to attempt to accomplish this. However, if they cannot do it or if they adamantly refuse to quit, we do not deny them a surgical repair. This might also prevent them presenting with an incarcerated hernia that might need emergent repair, placing these patients at a much higher risk of recurrence and complications.

### 6.2. Laparoscopic Repair of Umbilical Hernias in Obese Patients

The laparoscopic approach is another strategy to reduce recurrence and complications from UHR [60]. Guidelines from the EHS/AHS recommend laparoscopy for large defects and patients at risk of developing a SSI [2]. Laparoscopy for UHR in obese patients has been shown to reduce the rate of SSI (26.0% vs. 4.0%; *p* < 0.05) without affecting the rate of recurrence [89]. Laparoscopy was also found to be superior to tissue repair in another retrospective study [90]. In a separate study, UHR was undertaken laparoscopically, open with mesh, and open via tissue repair. No difference in the rate of recurrence was observed in the various techniques [60]. Today, there is no specific criteria for UHR via laparoscopy. Cost, expertise, and equipment availability remain a concern.

The laparoscopic technique most commonly employed for repair of an umbilical (or other ventral) hernia involves the intraperitoneal placement of a coated mesh to provide broad flat coverage of the hernia defect of at least 5 cm in each direction. This is often termed an intraperitoneal onlay mesh (IPOM) repair. Traditionally the hernia was reduced, but the fascial defect was not routinely closed. Many surgeons now advocate closure of all fascial defects using transfascial suture passer devices, or less commonly by suturing defects laparoscopically [91]. The mesh is generally fastened to the abdominal wall by circumferential rings of tacks made of absorbable plastic or permanent metals. The laparoscopic technique has the advantage of allowing placement of a larger mesh with broader defect overlap when compared to open mesh placement techniques. Other techniques include preperitoneal and retromuscular hernia repair, but these are far more commonly performed using robotic assistance [92].

Author’s Practice: In our experience, for many patients who come to clinic with an UH and suffer from class II or III obesity, if they do not want to proceed with bariatric surgery, we offer an elective repair. We inform them of the higher risk of recurrence and infection based on other studies, and if they want to proceed with repair, we provide that option to them. This is predicated on the fact that in our experience this patients are asked to lose weight, but are unable to do it only presenting to clinic at a later time with a higher weight or emergently with an incarceration.

If repair is offered laparoscopically to a patient with morbid obesity, it is critical to maximize mesh coverage to a minimum of 5 cm, not the standard of 3–5 cm in each direction. Our experience combined result from multiple retrospective studies around the globe indicate that primary umbilical hernia repair is feasible independent of obesity or hernia size. Recurrence rates in our veteran population did not differ significantly among patients of varying defect sizes or BMI categories. Our recurrence rates are compared to those reported in the literature and are within acceptable rates [47,48,49,53,66,69]. However, these observations are based on our institutional experience and others have shown different results. Therefore, these observations might not be applicable to other cohorts of patients.

### 6.3. Robotic Umbilical Hernia Repair

It is undeniable that robotic surgery is becoming a prominent strategy in the repair of abdominal wall hernias. The guidelines published in 2020 by the EHS/AHS did not even address robotics for UHR [2]. The robotic platform has led to significant innovation in the world of hernia surgery and several new techniques for ventral and umbilical hernia repair are being used with regularity in the United States and internationally. Like laparoscopy, a common robotic technique is the IPOM method. Some modifications include to directly suture the umbilical defect closed, including tacking down the umbilical stalk to improve the cosmetic appearance of the umbilicus. The mesh is also usually sutured in place circumferentially instead of using tacks for fixation.

There are other robotic techniques that all place mesh within or outside of the layers of the abdominal wall so that there is no direct interaction between bowel and mesh. These techniques include preperitoneal mesh placement, called the robotic transabdominal preperitoneal (TAPP) repair. In a robotic TAPP, the peritoneum is dissected away from the abdominal wall fascia so that a large piece of macroporous, uncoated mesh can be placed between the fascia and peritoneum. Other advanced robotic techniques include the robotic extended totally extraperitoneal (eTEP) approach, and the subcutaneous onlay approach (SCOLA) [93]. These approaches place mesh in the retromuscular and subcutaneous layers, respectively, and may be useful in patients with significant rectus diastasis where simultaneous diastasis plication is desired [94].

Author’s Practice: In our opinion, robotic TAPP or robotic IPOM should be considered the first-line robotic repair techniques for UH, with other more experimental techniques reserved for special circumstances. Data for the effectiveness and safety of robotic repair techniques for ventral hernias have generally shown similar outcomes to laparoscopic techniques with longer operative times and increased cost. Robotic repair is optimal for morbidly obese patients. It permits closure of the hernia defect to reduce the risk of a mesh invaginating into the hernia defect and possibly trapping abdominal contents with it. It also avoids surgeon fatigue from dealing with a thick abdominal wall. Finally, it has been the experience of our group that up to 45% of morbidly obese patients with umbilical hernias also have a concurrent epigastric hernia. Exposure of the epigastric abdominal wall is critical in this patient population. It is also important to note that our federal facility (a Veterans Administration Hospital) has three robots available for our group. We thus acknowledge that this might represent some inherent bias towards offering this repair to our veterans. Operating room times and cost are still a major concern for this “minor operation”. However, outside of the VA, access limitations and const remain a major concern for robotic UHR [94].

## 7. Conclusions

A “gold-standard” for the management of umbilical hernias must be personalized in the 21st Century. Three important elements of this personalized approach include the expertise of the surgeon, the resources available in the hospital, and the preferences of the patient. For instance, our approach to an older veteran with a substantial number of comorbid conditions and a small, asymptomatic UH is WW. For a patient in an LMIC where the hospital lacks resources and mesh as well as general anesthesia are limited, an open tissue repair under regional anesthesia might be the only possible approach. A patient with a recurrent hernia presenting to a hospital with high laparoscopic or robotic volume might benefit from a minimally invasive approach. None of these strategies are competing but complementary and tailored to system-based practices. A general surgeon should be familiar with these techniques to properly advise patients. It is important to discuss risks and benefits of each approach, including operative room times and cost, such that shared decision making takes place. Smoking cessation and weight loss should be recommended to patients prior to elective repair, but patients who adamantly refuse or are unable should not be denied care. The observations in this perspective show that there is strong evidence by meta-analyses, RCTs, and strong retrospective studies, which have resulted in sound guidelines for the management of UH. However, our intuitional experience combined with retrospective studies from others underscore that deviation from guidelines might be undertaken with caution considering the patient population, experience of the surgeons, and institutional resources.
